# Assessment of Different Risk Factors Among Adult Cardiac Patients at a Single Cardiac Center in Saudi Arabia

**DOI:** 10.7759/cureus.11649

**Published:** 2020-11-23

**Authors:** Jamilah AlRahimi, Rouya Alattas, Hidaya Almansouri, Ghadah B Alharazi, Hani N Mufti

**Affiliations:** 1 Department of Cardiac Science, Ministry of National Guard Health Affairs, College of Medicine, King Saud bin Abdulaziz University for Health Sciences, King Abdullah International Medical Research Center, Jeddah, SAU; 2 Department of Medicine, Ministry of National Guard Health Affairs, King Saud Bin Abdulaziz University for Health Sciences College of Medicine, Jeddah, SAU; 3 Department of Medicine, King Abdullah International Medical Research Center, Jeddah, SAU; 4 Department of Cardiac Surgery, King Faisal Cardiac Center, King Abdullah Medical City, Jeddah, SAU; 5 Department of Medicine, King Saud Bin Abdulaziz University for Health Sciences, Jeddah, SAU

**Keywords:** coronary artery disease, diabetes, smoking, hypertension

## Abstract

Background

Cardiovascular disease (CVD) has remained the leading cause of death in the last 15 years and is one of the main health problems in Saudi Arabia. Our study aims to assess the prevalence of different CVD risk factors and correlate them among King Faisal Cardiac Center patients in King Abdul-Aziz Medical City in Jeddah, Saudi Arabia.

Methods

We conducted a cross-sectional study using a convenient sampling technique. Data were collected by interviewing adult patients admitted to King Faisal Cardiac Center and diagnosed with hemodynamically stable cardiac disease. We excluded patients with multiple medical conditions that contribute to acute mental disorders. The sample size was calculated to be 200 patients.

Results

Overall, 163 patients completed the survey. The majority of the participants (49.1%) were between 46-65 years of age, males, non-smokers, and had more than 11 children. Diabetes was found to be the most common risk factor (66.3%). Most participants had mild to moderate anxiety (63.8%) and depression (66.9%). Most of the patients (51.5%) have a high 10-year risk of developing CVD, followed by moderate and low risk (33.1% and 15.3%, respectively).

In our study, a high 10-year risk of CVD was significantly associated with age between 46-80 years with a p-value=0.002, male gender with a p-value=0.007, cigarette smoking with a p-value=0.031, and diabetes with a p-value=0.035.

Conclusion

The study demonstrated a high prevalence of the following CVD risk factors: age, male gender, immobility, obesity, diabetes, dyslipidemia, and hypertension. In addition, a significant association was found between high 10-year risk of CVD and age, gender, smoking, number of children, and diabetes with a p-value<0.05. No significant association was found in the other risk factors such as obesity, body mass index (BMI), immobility, caregiver, dyslipidemia, depression, and anxiety.

## Introduction

Cardiovascular diseases (CVDs) are a group of disorders that affect the heart and blood vessels [1]. CVD is the leading cause of death worldwide, with increasing mortality each year. In 2015, ischemic heart disease (IHD) and stroke accounted for approximately 15 million out of 56.4 million deaths worldwide [2]. Coronary heart disease is one of the main chronic illnesses in Saudi Arabia, and it is known as the third most common cause of hospital-based mortality [3]. The prevalence of CVD is due to several risk factors, including hypertension, hypercholesterolemia, obesity, tobacco smoking, positive family history, an unhealthy diet, and poor physical activity. These factors are influenced by behavioral, social, cultural, and economic factors [4]. Assessing these factors and their correlation with adult cardiac patients at King Faisal Cardiac Center in King Abdul-Aziz Medical City in Jeddah, Saudi Arabia, is the aim of this study.

A person’s health status plays a major role in their susceptibility to having a CVD. Obesity and being overweight, defined by the World Health Organization as abnormal or excessive fat that accumulates, present a health risk, and increase the risk of many chronic diseases. Obesity may be associated with hypertension, dyslipidemia, diabetes or insulin resistance, and elevated levels of fibrinogen and C-reactive protein; all of which increase the risk of CVD events [5-7]. Consequently, these chronic conditions greatly increase the chance of cardiovascular illness. Persistent hypertension is one of the risk factors for stroke, myocardial infarction (MI), heart failure, and arterial aneurysm. It is also a leading cause of chronic kidney failure [5]. Another health condition that affects the cardiovascular system is hypercholesterolemia. People with hypercholesterolemia are at higher risk of developing coronary artery disease.

CVD can be influenced by lifestyle. An unhealthy diet, minimal physical activity, and smoking have a great influence on the cardiovascular system health. Smoking increases the risk of CVD. The general mechanisms by which smoking results in cardiovascular events include the development of atherosclerotic changes, with a narrowing of the vascular lumen and induction of a hypercoagulable state, which creates a risk of acute thrombosis [8]. On the other hand, physical activity has a major positive impact on cardiovascular health. Exercise is vital to reduce the risk of heart disease. Walking at least 30 minutes each day at a vigorous pace (at least 4 km per hour) reduces heart disease risk by 30% [9-13].

Furthermore, a mental health assessment is essential to identify patients who are suffering from mental or behavioral disorders and tailor the management plan to improve their quality of life. Mental illness is defined as a condition that involves any abnormal changes in thinking, emotion, and/or behavior [14]. An independent association has been found between depression or anxiety and negative cardiac outcomes among patients suffering from cardiac diseases and acute cardiac events [14]. Also, it has been found that 15% of cardiac patients suffer from major depression syndrome, which is high as compared to the general population (4%-5%). Furthermore, the percentage of self-reported anxiety has increased in patients that experienced an acute myocardial infarction (20%-50%) [15]. Moreover, emotional support, social isolation, and lack of interpersonal social relations are important risk factors that have a strong impact on the incident and the progression of coronary artery disease. One study has shown that in women with a lack of social support, the arterial luminal diameter was found to be narrower [16].

A caregiver is a professional, family member, or friend that takes care of a patient and helps them with daily activities such as personal care, emotional support, as well as helping them with their medications [17]. Caregiving is another factor that is believed to improve the outcome of cardiac patients [18]. This research will include caregiving as a factor that is studied to investigate its effect on cardiac outcome. Certain aspects related to the caregiver will be studied such as the relationship of the caregiver with the patient, the tasks that the caregiver performs, such as taking care of medication and personal care, the dependence of the patient on the caregiver, and how frequently the caregiver takes care of the patient.

## Materials and methods

This is a cross-sectional study conducted in King Faisal Cardiac Center at King Abdul-Aziz Medical City in Jeddah, Saudi Arabia. We targeted adult patients of both genders who were admitted in the cardiac ward (non-critical care area) and clinically diagnosed with cardiac disease that does not require any hemodynamical monitoring or support. Patients with multiple medical conditions that contribute to acute mental disorders, such as thyroid disease in the acute stage, end-stage renal disease, or any similar conditions, were excluded. The sample size for this study was calculated using the “Openepi” software, www.openepi.com/menu/OE_menu/htm, with a confidence level of 95%, a confidence interval of 6.5%, and a target population of 2000. The target sample was calculated to be 200 patients over the study time frame (1 year after the approval), which was estimated from the admitted cardiac patients at King Faisal Cardiac Centre over the last 14 months. However, only 163 patients were included. The sample was collected by a non-probability convenient sampling technique.

Data collection process

This study used a questionnaire that included questions about demographics, Patient Health Questionnaire-9 (PHQ-9) and Generalized Anxiety Disorder-7 (GAD-7), and a questionnaire that included questions about other cardiac risk factors, such as smoking and factors related to the caregiver, to assess patients’ demographic data. The demographic questions included questions about age, gender, marital status, number of children, and economic status. The depression and anxiety questionnaires, PHQ-9 and GAD-7, resulted in a scored number according to the patients’ answers and determined the severity and the level. PHQ-9 scores of 5, 10, 15, and 20 represent mild, moderate, moderately severe, and severe depression respectively. The GAD-7 is a seven-item instrument used to briefly assess for mild, moderate, and severe anxiety with cut-off points of 5, 10, and 20, respectively. The questionnaires are internationally validated and are very reliable sources to assess the frequency and level of symptoms of anxiety and depression. The third questionnaire used for the assessment of health-related risk factors was modified and translated into an Arabic version of the Healthy Heart Questionnaire (HHQ-GP-1), which is recommended by the University of Colorado Denver. The cardiac patients who were willing to participate were interviewed and the questionnaires were administered.

The main outcome of the study was to identify the correlation between selected factors related to health, lifestyle, and psychosocial status in stable adult cardiac patients. The study factors were divided into selected factors related to health, lifestyle, and psychosocial status. The selected factors related to health and lifestyle included exercise and smoking. In addition to that, the psychosocial factors included depression and generalized anxiety disorder. The grouping variables of this study were stable cardiac patients with different severities of the disease. They were categorized into mild, moderate, and severe cardiac diseases. The estimation of the risk of the cardiovascular outcome was identified by the Framingham risk assessment tool. The patient data were accessed through the health informatics system “Best Care.”

Data analysis

Data collected were entered into a datasheet in Microsoft Excel, then transferred to SPSS version 22 (IBM Corp, Armonk, NY) for analysis. To ensure the accuracy of the data analysis, Optical Character Recognition (OCR) Microsoft OneNote software (Microsoft Corporation, Redmont, WA) was used to reduce some of the inaccuracies. Quantitative data were presented by mean and standard deviation while qualitative data were presented by frequency and percentage. To compare qualitative variables, the chi-square test and p-value <0.05 were used to determine statistical significance. 

## Results

The initial number of study subjects was 200, however, a total of 163 patients completed the survey (response rate 81.5%) in this ongoing study. Most of the patients in this study were 46-65 years of age (49.1%). The majority of the patients were male (60.1%); 84.0% of all participants were married. Most of the participants (47.2%) have between six and 10 children. While 13.5% have more than 11 children. Besides, approximately 80% (n=131) of the patients had a caregiver with them, where 50% of those patients were independent and did not rely on their caregiver. More than half of the patients in this study were non-smokers (60.7%). A large number of participants (58.9%) spend five hours or more sitting during the day and 71.2% spend zero hours exercising (Table 1).

**Table 1:** Demographic and other characteristics

Age	N (%)
18-45	25 (15.3)
46-65	80 (49.1)
66-80	50 (30.7)
>80	8 (4.9)
Gender	
Male	98 (60.1)
Female	65 (39.9)
Marital status	
Single	8 (4.9)
Married	137 (84.0)
Widowed	13 (8.0)
Divorced	5 (3.1)
Number of children	
0-5	63 (38.7)
6-10	77 (47.2)
11 and more	22 (13.5)
Caregiver	
Yes	130 (79.8)
No	33 (20.2)
Employee	
Yes	39(23.9)
No	124(76.1)
Smoking	
Cigarettes	45 (27.6)
Shisha	11 (6.7)
Never	99 (60.7)
Quit	8 (4.9)
Exercise (hours/day)	
0	116 (71.2)
1-3	19 (11.7)
4-7	28 (17.2)
Number of hours seated	
1-4	67 (41.1)
5 and more	96 (58.9)
BMI	
Underweight < 18.5 kg/m2	3 (1.8)
Normal 18.5-25 kg/m2	27 (16.6)
Overweight 25-30kg/m2	46 (28.2)
Obese >30kg/m2	87 (52.4)

Diabetes (66.3%) was found to be the most common risk factor among participants followed by obesity, with BMI > 30 kg/m^2^ (52.4%), dyslipidemia (47.9%), and hypertension (75.5%) (Table 2).

**Table 2:** Health-related characteristics

	N (%)
Diabetes	108 (66.3)
Dyslipidemia	78 (47.9)
Hypertension	123 (75.5)

Furthermore, the prevalence of mild to moderate depression and anxiety in the participants of this study was high; 109 patients (66.9%) had mild-moderate depression and 104 patients (63.8%) had mild-moderate anxiety (Table 3).

**Table 3:** Mental health characteristics

Depression	N (%)
No depression	20 (12.3)
Mild-moderate	109 (66.9)
Severe-very sever	34 (20.9)
Anxiety	
No anxiety	47 (28.8)
Mild-moderate	104 (63.8)
Severe-very severe	12 (7.4)

Most of the patients in this study (51.5%) were found to have a risk of coronary artery disease (CAD) within 10 years >20%, which is measured by the Framingham risk score that estimates the 10-year cardiovascular risk of an individual. The following factors were used to calculate the risk: age, cholesterol level, smoking, diabetes, and hypertension. In addition, 54 patients (33.1%) were found to have moderate risk (10%-20%) of developing CAD within 10 years. On the other hand, 25 patients (15.3%) were found to have low risk.

The chi-square test of independence was performed to determine whether there is a relationship between cardiac risk factors and the risk of IHD. The risk of IHD was associated with age, gender, smoking, number of children, and diabetes with a p-value that is less than 0.05. The chi-squared test was performed to test whether there is an association between age and the risk of IHD, which was found to be significant; p-value=0.002. Results showed that patients aged 18-45 and patients above 80 were most likely to have a mild-moderate risk of IHD while patients aged 46-80 were more likely to have a high risk of IHD. Also, there is an association between gender and risk with a p-value of 0.007. Males were more likely to have a high risk of IHD, where 60% of the male patients had a high risk and 60.1% of female patients had a low to moderate risk. Another significant association was found between smoking and the risk of IHD with a p-value of 0.031. Most cigarette smokers (64.4%) fell within the high-risk category, and most of the non-smokers (57.6%) fell within the mild-to-moderate risk category. Besides, the number of children the patient has was associated with high risk and a p-value of 0. 011. Patients with a higher number of children were more likely to have a high risk of IHD; 72.7% of patients with more than 10 children had a high risk according to the Framingham score. Most diabetic patients (57.4%) have a high risk of IHD while (42.6%) have mild to moderate risk with a p-value of 0.035. Table 4

**Table 4:** Association between risk factors and the risk of developing cardiovascular disease

	Severity		p-value
	Mild to moderate n (%)	High n (%)	
Age			0.002
18-45	20 (80)	5 (20)	
46-65	32 (40)	48 (60)	
66-80 >80	21 (42) 6 (75,1)	29 (58) 2 (25)	
Gender			0.007
Male	39 (39.8)	59 (60.2)	
Female	40 (61.5)	25 (38.5)	
Number of children			0.011
0-5	39 (61.9)	24 (38.1)	
6-10	34 (44.2)	43 (55.8)	
≥11	6 (27.3)	16 (72.7)	
Smoking			0.031
Cigarettes	16 (35.6)	29 (64.4)	
Shisha	4 (36.4)	7 (63.6)	
Never	57 (57.6)	42 (42.4)	
Quit (cigarettes)	2 (25)	6 (75)	
Diabetes	46 (42.6)	62 (57.4)	0.035

## Discussion

The purpose of this study was to assess the prevalence of risk factors that contribute to the development of IHD. The study aimed to assess different health, psychological, and social factors and their effect on the risk of developing a CVD.

CVDs were associated with the following risk factors in literature: hypertension, smoking, obesity, and poor physical activity. However, in our study, out of these factors, an association was only found between smoking and the risk of IHD

Moreover, the Framingham score, which is used to assess the 10-year risk of developing coronary heart disease, accounts for smoking as one of the IHD risk factors. Smoking provokes atherosclerotic changes that lead to the narrow vascular lumen, hypercoagulable state, and, eventually, thrombosis [9]. Our results show a significant association between smoking and an increase in CVD risk. Furthermore, diabetes is a well-known risk factor for CVD, as it can affect both small and large vasculature. Some guidelines consider diabetes mellitus equivalent to coronary heart disease when it comes to the 10-year risk of CVD (≥20%) [19-22]. In our study, an association was found between diabetes and the 10-year risk of IHD with a p-value of 0.035.

According to Maas A and his group, CVDs develop later in women than in men, usually seven to eight years later [23-24]. In our study, an association was found between gender and the 10-year risk of IHD, where men were more likely to have a high risk of CVD. In another study, older age was associated with a higher risk of CVD [22-28]. In our study, the majority of patients aged 40-80 were at a high risk of CVD. However, patients above the age of 80 were at a low-moderate risk. A possible reason for that is that there were only eight patients above the age of 80, making it difficult to generalize the results. Many patients were not cooperative and refused to answer the questionnaires or decided to stop the interviews abruptly. Also, many of the admitted patients were too ill to answer the questions. Also, many of the patients are constantly readmitted to the inpatient ward, therefore reducing the number of new patients from whom data can be collected from. These factors contribute to the small sample size. Another limitation of the study was the possibility of recall bias, especially that some of the questions depended on their ability to remember the answers.

Even though the literature showed that there is an independent association between depression and anxiety and negative cardiac outcome [6], our results did not support that, as they were insignificant. A possible reason for this is that most patients were usually accompanied by family members, therefore, some of the patients may not have felt comfortable answering questions related to depression and anxiety. Most of the patients in this study were accompanied by caregivers; this can be related to the strong family bonds that are a part of the Saudi Arabian culture; and as reported by Mosca L et al, having a caregiver improves patient adherence to medication and the follow-up and nutrition plan that improves the patient outcome [29-30]. A significant association was found between the number of children a patient has and the risk of CVD, where the risk increases with the number of children. However, there was no literature found to compare this finding too.

A possible reason for the insignificance of the other results is that the study was only carried in a single center with a small sample size. In addition to that, only 80% of the expected sample size was collected. Furthermore, with relevance to physical activity, only a minority of the patients exercise or follow an active lifestyle, also making it difficult to generalize the data.

Limitation

Other well-known risk factors were not found to be associated with the risk of CVD, and this can be mainly attributed to the small sample size, recall bias, and the study being carried out in a single-center only.

## Conclusions

In our study, the risk of developing CVD was significantly associated with some well-known risk factors (namely, increased age, male gender, smoking, and diabetes type II). Interestingly, we found that patients with a large number of children had a risk factor for CVD. The increase of CVD risk with the increase in the number of children might be due to the increased stress related to larger family burdens and responsibilities that might have an indirect impact on lifestyle choices (like smoking or unhealthy diet). To our knowledge, this is the first study that highlights the number of children as a risk factor for CVD. Future studies should investigate the impact of family size and life stressors on the development of CVD.
